# Hip Fracture Intervention Study for Prevention of Hypotension Trial: a Pilot Randomized Controlled Trial

**DOI:** 10.1213/XAA.0000000000001891

**Published:** 2025-01-06

**Authors:** Matthew S. Luney, Stuart M. White, Iain K. Moppett

**Affiliations:** From the * Nuffield Department of Orthopaedics, Rheumatology and Musculoskeletal Sciences, University of Oxford, Oxford, UK; † Department of Anaesthesia, Brighton and Sussex University Hospitals NHS Trust, Brighton, UK; ‡ Anaesthesia and Critical Care Section, Academic Unit of Injury, Inflammation and Repair, University of Nottingham, Nottingham, UK.

## Abstract

**BACKGROUND:**

Hypotension during anesthesia for surgery for hip fracture is common and associated with myocardial injury, stroke, acute kidney injury, and delirium. We hypothesized that maintaining intraoperative blood pressure close to patients’ preoperative values would reduce these complications compared to usual care.

**METHODS:**

A pilot feasibility patient- and assessor-blinded parallel group randomized controlled trial. People with unilateral hip fracture aged ≥70 years with capacity to give consent before surgery were eligible. Participants were allocated at random before surgery to either tight blood pressure control (systolic blood pressure ≥80% preoperative baseline and mean arterial blood pressure ≥75 mm Hg) or usual care. Feasibility outcomes were protocol adherence, primary outcome data completeness, and recruitment rate. The composite primary outcome was myocardial injury, stroke, acute kidney injury or delirium within 7 days of surgery.

**RESULTS:**

Seventy-six participants were enrolled, and 12 withdrew before randomization. Sixty-four participants were randomized, 30 were allocated to control, and 34 to intervention. There was no crossover, all 64 participants received their allocated treatment, primary outcome was known for all participants. The composite primary outcome occurred in 14 of 30 participants in the control group compared with 23 of 34 participants in the intervention group (*P* = .09), relative risk 1.45 (95% confidence interval [CI], 0.93–2.27).

**CONCLUSIONS:**

A randomized controlled trial of tight intraoperative blood pressure control compared to usual care to reduce major postoperative complications after fractured neck of femur surgery is possible. However, the data would suggest a large sample size would be required for a definitive trial.

**KEY POINTS**

- **Question:** Is tight intraoperative blood pressure control to reduce postoperative morbidity after hip fracture surgery feasible and effective compared to usual care?
- **Findings:** This multicenter pilot randomized controlled trial successfully delivered the intervention to all randomized participants, completed primary outcome follow-up for all participants and found the composite primary outcome myocardial injury, stroke, acute kidney injury and delirium was not significantly lower after tight blood pressure control.
- **Meaning:** Maintaining intraoperative blood pressure close to individual’s preoperative value is feasible; however, the effect on postoperative complications was not significant.

Depending on its definition, hypotension occurs in approximately 30% of people during surgical hip fracture repair.^1,2^ Postoperative mortality has been shown to be significantly higher among patients whose intraoperative mean arterial pressure (MAP) falls <55 mm Hg compared to those whose lowest MAP remained above this threshold,^3–6^ suggesting a plausible causal link between hypotension during surgery and postoperative complications. However, recent studies question whether brief periods of hypotension during anesthesia for hip fracture are detrimental. Guidance promotes avoidance of hypotension; precise thresholds and target intraoperative blood pressures targets however remain unresolved.^7–9^

Strict blood pressure control as part of protocolized treatment aimed at maintaining cerebral oxygenation has been found to significantly reduce the incidence of mild and moderate postoperative cognitive dysfunction,^10–12^ myocardial injury, and acute kidney injury (AKI) after hip fracture.^13,14^ Each of these postoperative complications is known to be independently associated with poorer outcomes (increased mortality and institutionalization, prolonged length of stay, and delayed return to mobility) among people with hip fracture. Tight blood pressure control has been shown in the INPRESS trial to reduce cardiovascular and renal morbidity after major abdominal surgery.^15^

We hypothesized that intraoperative hypotension may result in hypoperfusion of the brain, heart, and kidneys in older, frailer hip fracture patients with comorbidities, producing measurable postoperative alterations in organ function and resulting in poorer outcomes. Given the extant guidance, it is unclear whether a trial of tight blood pressure control is feasible. We have therefore performed a pilot randomized controlled trial comparing protocolized tight intraoperative control of blood pressure with standard treatment, using a composite of defined cardiovascular, renal and delirium scores within 7 days as the primary outcome, and postoperative mortality, fitness for hospital discharge, return to baseline mobility level and return to independent living as secondary outcomes. We report here the outcomes of our pilot study with the aim to establish feasibility of the protocol to conduct the definitive study. The definitive trial would test the hypothesis that tight intraoperative blood pressure control using vasopressors and fluids during hip fracture surgery under either general or spinal anesthesia improves outcomes compared with standard treatment.

## METHODS

Ethical approval for this study was given by the East Midlands – Nottingham 1 Research Ethics Committee (REC reference 16/EM/0036). The trial was prospectively registered on ISRCTN before enrollment of the first participant (ISRCTN89812075, principle investigator: I. Moppett, date of registration: August 30, 2016). Written informed consent was obtained for each participant at enrollment and reconfirmed at each postoperative visit. The relevant Consolidated Standards of Reporting Trials (CONSORT) guideline was followed and the checklist for reporting a pilot trial^16^ is available as a supplementary document.

This is a parallel group randomized interventional pilot trial with investigators and participants blinded to treatment allocation. The participants were recruited in 3 centers, all NHS hospitals in England, UK: Queens Medical Centre Nottingham, Royal Sussex County Hospital Brighton, and Royal Surrey County Hospital Guildford. The trial protocol has been published.^17^ There were no protocol changes made during the pilot trial.

Patients admitted for management of hip fracture were screened on inpatient trauma wards in secondary care settings before surgery. The enrollment of participants was performed by research nurses preoperatively. Data were collected preoperatively and postoperatively on these same wards. Intraoperative blood pressure data were recorded in the operating theater via screenshots/ electronic export of the monitor output. Anesthetic chart review of intraoperative blood pressure was performed where any intraoperative blood pressure screenshot data were missing.

The eligibility criteria for inclusion in the study were unilateral hip fracture, aged over 70, and able to give consent. Exclusion criteria were patients due to undergo total hip arthroplasty, patients with peri-prosthetic fractures, patients without capacity, and patients with preoperative elevated troponin (measured for clinical reasons).

### Intraoperative Management

The intervention arm was tight intraoperative blood pressure control using standard anesthetic drugs in addition to usual care. The control arm received usual care according to national UK standards (NICE, BOAST, AAGBI, and Best Practice Tariff).^18–21^

Participants randomized to the intervention “tight blood pressure control” arm received active treatment as required to maintain both systolic blood pressure ≥80% of baseline preoperative value (defined as the mean of 2 readings taken at least 5 minutes apart after the patient has arrived in the induction room for anesthesia) and MAP ≥75 mm Hg throughout the operation. The order and doses of treatments to achieve this were determined by the clinical scenario at the discretion of the anesthetist responsible for the patient. The treatments included: intravenous fluid boluses, ephedrine and the sympathomimetic metaraminol (with comparable effects to phenylepherine but more widely used in UK practice). Monitoring of blood pressure was performed at least every 2.5 minutes in the intervention group, or using continuous noninvasive blood pressure monitoring (Nexfin, BMEYE). Frequency of blood pressure monitoring in the control group was at the responsible anesthetists’ discretion but at least every 5 minutes in line with standard practice. Except for the tight blood pressure intervention outlined above participants in the control arm received the same care as the intervention arm in all other respects.

Randomization on a one-to-one basis was provided by security sealed sequentially numbered opaque envelopes. The sequence generation and preparation of the envelopes was performed by an individual from the administrative staff of Nottingham University Hospitals research department who was otherwise not involved in the trial. The randomization was computer generated in blocks using a random seed known only to the aforementioned individual independent from the investigators. Randomization was stratified by the intended mode of anesthesia (general anesthesia via spinal anesthesia) and Nottingham Hip Fracture Score (NHFS) (≤4 vs ≥5). The sequence remained unknown until data lock. The timing of patient randomization was in the anesthetic room before induction of anesthesia where the anesthetist responsible for that patient would open the sealed envelope which detailed which treatment allocation to follow.

The responsible anesthetist was aware of the treatment allocation. All other staff including investigators were blinded to this information until data were locked. Participants were blinded to the treatment allocation. From a participant perspective the treatments in each group were indistinguishable from each other such that participants could not have been aware of their allocation; the differences in treatment do not result in symptoms or conditions that would indicate to the participant nor investigators which treatment group that participants were allocated to.

### Study Outcomes

Feasibility outcomes were protocol adherence, primary outcome data completeness, and recruitment rate.

The primary clinical outcome was a composite of presence of defined cardiovascular, renal, and cerebral morbidity within 7 days of surgery: myocardial injury, stroke, AKI, or delirium. These major postoperative complications were defined according to European Perioperative Clinical Outcomes (EPCO) definitions.^22^

The secondary outcomes were presence or absence of each of defined cardiovascular, renal and delirium morbidity within 7 days of surgery, 5-day postoperative mortality, 30-day postoperative mortality, quality of life at 30-days (EQ-5D), operation to fit-for-discharge time, operation to up-and-walk time, proportions returning to preoperative place of residence, and prevalence bone cement implantation syndrome.

### Statistical Analysis

The planned sample size of 75 participants was chosen to test trial feasibility. Sample size calculations for the definitive trial have already been fully described elsewhere.^17^ Briefly, major organ dysfunction after hip fracture surgery has been shown to occur in 20% patients. Taking an absolute event rate reduction from 20% to 15%, then 1250 participants would need to be randomized per group (α = 0.05 and β = 0.1). Similar effect sizes have been demonstrated before in perioperative outcomes after hip fracture surgery research.^13^

Feasibility outcomes are described using descriptive statistics. χ^2^ tests are used for proportional data including the primary composite outcome and secondary outcomes. Mortality data are reported as binary outcomes at 5 days and 30 days after surgery and using survival analysis with Cox-regression up to 1 year after surgery. Analysis was performed using R Version 4.2.1.^23^

## RESULTS

### Recruitment

**Figure 1.**
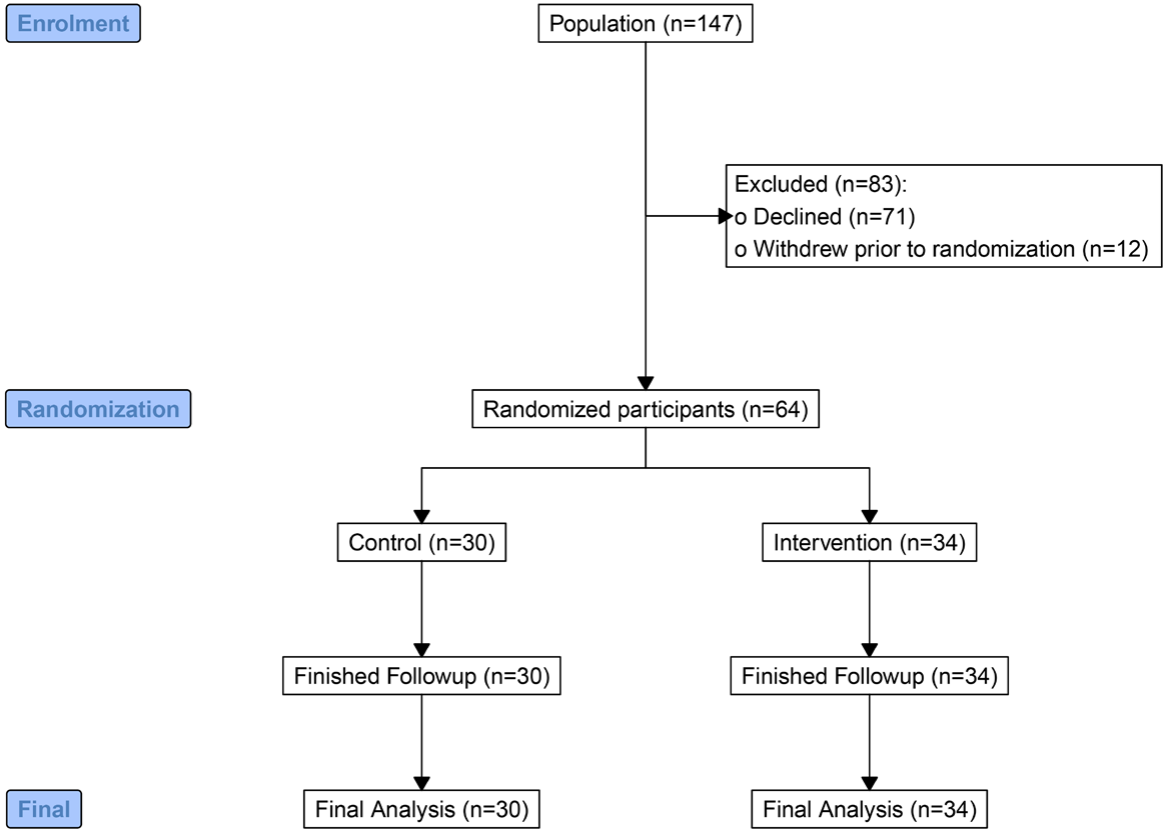
CONSORT diagram of participant enrollment, allocation and follow-up. CONSORT indicates Consolidated Standards of Reporting Trials.

Between December 2016 and October 2018, 147 participants were approached across the 3 trial sites. Seventy-six (52%) participants consented, 12 of whom withdrew from the trial before randomization. The recruitment rate was 3.5 participants per month. Sixty-four participants were randomly allocated to either control (n = 30) or intervention (n = 34) and analyzed below. Participant flow is shown in the CONSORT diagram (Figure 1). The final participant follow-up was completed on October 12, 2019. Analysis of data was delayed due to the impact of the COVID pandemic. Follow-up for the primary outcome was completed for all 64 randomized participants (100%). Baseline participant characteristics and anesthetic techniques were closely matched (Table 1).

**Table 1.** Baseline Characteristics of Trial Participants

Characteristic	Overall (n = 64)^a^	Usual care (n = 30)^a^	Tight blood pressure (n = 34)^a^
Age (y)	84 ± 7	83 ± 6	84 ± 7
Sex			
Female	48 (75%)	25 (83%)	23 (68%)
Male	16 (25%)	5 (17%)	11 (32%)
Operation			
DHS	15 (23%)	4 (13%)	11 (32%)
Hemiarthroplasty	22 (34%)	12 (40%)	10 (29%)
Cannulated screws	6 (9.4%)	3 (10%)	3 (8.8%)
Femoral nail	8 (12%)	4 (13%)	4 (12%)
Not recorded	13 (20%)	7 (23%)	6 (18%)
Usual residence			
Home	54 (84%)	26 (87%)	28 (82%)
Sheltered	5 (7.8%)	2 (6.7%)	3 (8.8%)
Not recorded	5 (7.8%)	2 (6.7%)	3 (8.8%)
Number of major comorbidities			
0	10 (16%)	2 (6.9%)	8 (24%)
1	13 (21%)	8 (28%)	5 (15%)
2	14 (22%)	8 (28%)	6 (18%)
3	11 (17%)	8 (28%)	3 (8.8%)
4	9 (14%)	3 (10%)	6 (18%)
5	2 (3.2%)	0 (0%)	2 (5.9%)
6	4 (6.3%)	0 (0%)	4 (12%)
Malignancy in last 20 y			
Yes	14 (22%)	7 (23%)	7 (21%)
No	47 (73%)	21 (70%)	26 (76%)
Not completed	1 (1.6%)	1 (3.3%)	0 (0%)
Not recorded	2 (3.1%)	1 (3.3%)	1 (2.9%)
Mobility score^24^	6.65 ± 2.22	6.54 ± 2.32	6.74 ± 2.16
Frailty score^25^	6.9 ± 4.8	7.1 ± 4.5	6.8 ± 5.1
Preoperative SBP (mm Hg)	143 ± 23	148 ± 29	138 ± 16
Preoperative DBP (mm Hg)	72 ± 12	71 ± 14	72 ± 11
Mode of anesthesia			
General anesthesia	33 (52%)	15 (50%)	18 (53%)
Spinal anesthesia	31 (48%)	15 (50%)	16 (47%)
Induction agent			
Propofol	26 (79%)	14 (93%)	12 (67%)
Etomidate	3 (9.1%)	0 (0%)	3 (17%)
Sevoflurane	4 (12%)	1 (6.7%)	3 (17%)
Neuromuscular blocking agent			
None used	13 (39%)	8 (53%)	5 (28%)
Atracurium	7 (21%)	2 (13%)	5 (28%)
Rocuronium	12 (36%)	4 (27%)	8 (44%)
Drug name unknown	1 (3.0%)	1 (6.7%)	0 (0%)
Airway device used			
Own	0 (0%)	0 (0%)	0 (0%)
Supraglottic airway	13 (39%)	8 (53%)	5 (28%)
Tracheal tube	19 (58%)	6 (40%)	13 (72%)
Not recorded	1 (3.0%)	1 (6.7%)	0 (0%)
Depth of anesthesia monitoring used	7 (21%)	3 (20%)	4 (22%)
Dose of spinal anesthetic (0.5% bupivacaine in mL)	1.8 ± 0.5	1.8 ± 0.5	1.9 ± 0.5
Regional block used			
Femoral	22 (34%)	10 (33%)	12 (35%)
Fascia Iliaca	21 (33%)	11 (37%)	10 (29%)
3 in 1	0 (0%)	0 (0%)	0 (0%)
None	21 (33%)	9 (30%)	12 (35%)
Laboratory values on admission			
Hemoglobin (g.L^–1^)	123 ± 17	122 ± 16	124 ± 18
White cell count (µL^–1^)	10.9 ± 3.2	11.1 ± 3.4	10.8 ± 3.1
Platelet count (µL^–1^)	251 ± 70	259 ± 69	245 ± 72
Creatinine (µmol.L^–1^)	89.7 ± 50.1	87.4 ± 50.7	91.8 ± 50.3
Sodium (mmol.L^–1^)	136.0 ± 3.4	135.1 ± 3.9	136.8 ± 2.7
Potassium (mmol.L^–1^)	4.2 ± 0.6	4.1 ± 0.7	4.3 ± 0.5
eGFR (mL.min^–1^)	62.0 ± 22.1	64.1 ± 22.9	60.1 ± 21.6
Urea (mmol.L^–1^)	8.2 ± 4.1	8.0 ± 4.3	8.4 ± 4.0

Abbreviation: eGFR, estimated glomerular filtration rate.

a Mean ± SD; n (%).

### Feasibility Outcomes

**Figure 2.**
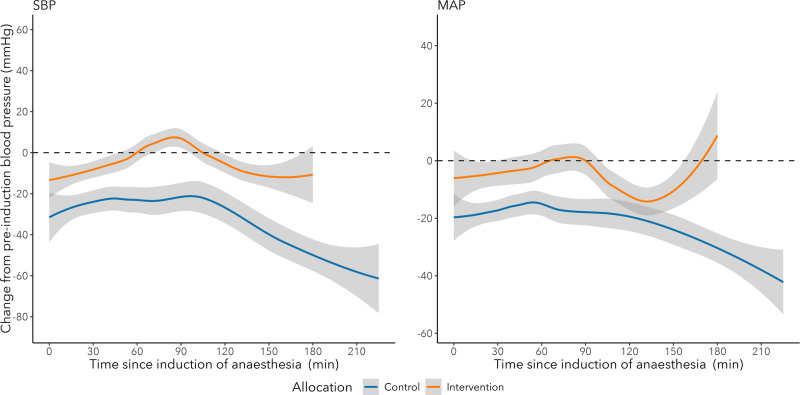
Relative change in blood pressure during hip fracture surgery for participants allocated to tight blood pressure control (orange) or usual care (blue). Mean pressures by group allocation are shown as solid lines. MAP indicates mean arterial pressure; SBP, systolic blood pressure.

Primary outcome data were obtained for all randomized participants (n = 64). The mean data completeness for measurement of all components of the composite primary outcome across the 7 day follow-up was 91.2%. One participant was missing intraoperative blood pressure data due to an IT failure. There was evidence of separation of the 2 groups by allocation (Figure 2 and Supplemental Digital Content 1, Figure S1, http://links.lww.com/AACR/A533). Our trial processes were unable to capture sufficient reliable data on participants’ operation to fit-for-discharge time, operation to up-and-walk time, nor the proportions returning to preoperative place of residence.

The median nadir in systolic blood pressure and nadir mean arterial pressure did not differ between control and intervention groups (Supplemental Digital Content 1, Figure S2, http://links.lww.com/AACR/A533). However, the median absolute times below target blood pressure (SBP within 80% baseline and MAP ≥75 mm Hg) were longer for the control group (Supplemental Digital Content 1, Table S1, http://links.lww.com/AACR/A533 and Supplemental Digital Content 1, Figure S3, http://links.lww.com/AACR/A533).

After adjusting for length of operation the duration below target remained longer for the control group. In the control group, the mean duration of systolic blood pressure <80% baseline was 24 min.h^–1^ compared to 13 min.h^–1^ in the intervention group. The mean duration of MAP <75 mm Hg was 18.8 min.h^–1^ in the control group and 12.5 min.h^–1^ in the intervention group.

The proportion of participants treated with metaraminol was lower in the control group (15/30, 50%) than in the intervention group (24/34, 71%), versus whereas ephedrine use was more common in the control group than in the intervention group (16/30, 53% vs 13/34, 38%) (Table 2 and Supplemental Digital Content 1, Figure S4, http://links.lww.com/AACR/A533). Ten participants did not receive vasoactive drugs (6 in control group, 4 in intervention group) (Supplemental Digital Content 1, Table S2, http://links.lww.com/AACR/A533). The volumes of intravenous fluids did not differ between the groups (Supplemental Digital Content 1, Table S3, http://links.lww.com/AACR/A533).

**Table 2.** Vasoactive Drug Administration By Treatment Allocation

Total dose	Overall (n = 64)^a^	Usual care (n = 30)^a^	Tight blood pressure (n = 34)^a^
Metaraminol (mg)	1.00 (0.00–2.50 [0.00–14.50])	0.05 (0.00–1.38 [0.00–14.00])	1.50 (0.00–3.00 [0.00–14.50])
Ephedrine (mg)	0 (0–7 [0–42])	5 (0–11 [0–42])	0 (0–6 [0–24])

Abbreviation: IQR, interquartile range.

a Median (IQR [minimum–maximum]).

### Clinical Outcomes

The composite primary outcome (myocardial injury, stroke, AKI or delirium within 7 days) occurred in 14/30 participants in the control group compared with 23/34 participants in the intervention group (*P* = .09) (Table 3) an absolute risk difference of 21%. The relative risk of the primary outcome was 1.45 (95% confidence interval [CI], 0.93–2.27).

**Table 3.** Effects of Tight Blood Pressure Control on Cardiovascular, Renal, and Cerebral Morbidity After Hip Fracture Surgery

Outcome	Usual care (n = 30)^a^	Tight blood pressure (n = 34)^a^	*P* value^b^
Composite primary outcome: cardiovascular, renal, and cerebral morbidity	14 (47%)	23 (68%)	.09
Myocardial injury	12 (40%)	20 (59%)	.13
Acute kidney injury	5 (17%)	6 (18%)	.92
Stroke or TIA	1 (3.3%)	0 (0%)	.47
Delirium	2 (6.7%)	6 (18%)	.26
5-d mortality	0 (0%)	0 (0%)	n/a
30-d mortality	0 (0%)	1 (2.9%)	>.99

Abbreviation: TIA, Transient ischemic attack.

a n (%).

b Pearson’s χ^2^ test.

**Figure 3.**
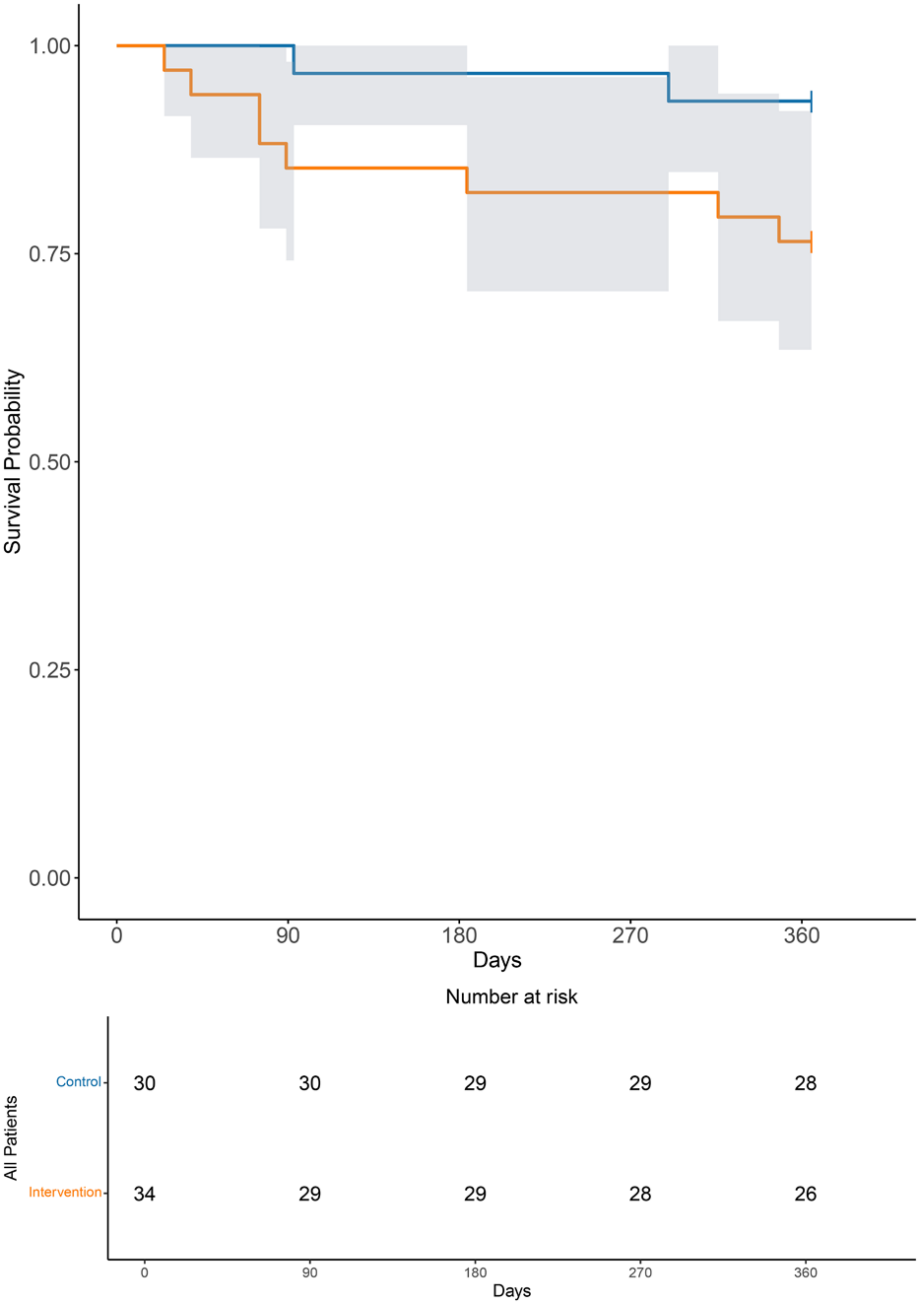
Kaplan-Meier plot for 1 y survival for participants allocated to tight blood pressure control (orange) or usual care (blue).

There were no significant differences for any of the secondary outcomes (Table 3 and Supplemental Digital Content 1, Tables S4 and S5, http://links.lww.com/AACR/A533). Ten participants (15.6%) died within 1 year of enrolling in the study (Figure 3). The median (interquartile range, IQR [range]) quality-of-life utility scores from the EQ-5D questionnaire at 30 days were 0.656 (0.283–0.710 [–0.429 to 1.000]) and 0.691 (0.374–0.771 [-0.239–1.000]) for the control and intervention groups, respectively. EQ-VAS scores were comparable between groups (Supplemental Digital Content 1, Figure S5, http://links.lww.com/AACR/A533).

There were 35 adverse events and 4 serious adverse events, none of which were assessed as related to the intervention (Supplemental Digital Content 1, Table S6, http://links.lww.com/AACR/A533). The most common adverse events were postoperative hypotension (n = 9/64) and anemia (n = 8/64).

## DISCUSSION

This pilot study demonstrated that a randomized controlled trial of tight blood pressure control during anesthesia for hip fracture surgery is achievable and that our target recruitment was met. Treatment adherence among anesthetists was good and there was separation in “time within target range” between groups.

There is lack of agreement on what blood pressure to target intraoperatively.^26,27^ Furthermore, the clinical benefit of individualized blood pressure management for hip fracture surgery remains unproven.^9^ There is conflicting evidence for the benefit or harm of interventions to target intraoperative hemodynamics, with trials demonstrating no effect, benefit and harm.^28–30^

With respect to achieving tight blood pressure control clinicians were better at maintaining target intraoperative mean arterial pressure than systolic blood pressure. Mean lowest SBP and MAP values between groups were similar suggesting that even in the usual care group anesthetists have notional prespecified blood pressure values of their own below which they will avoid (Supplemental Digital Content 1, Figure S2 http://links.lww.com/AACR/A533), or that it is technically challenging to prevent brief periods of hypotension. The work from Kluger et al^31^ suggests that this may not be clinically important.

Randomization for anesthesia-delivered interventions in the emergency surgical population is challenging. Postponement of procedures to later than the expected surgery time is common. Attrition (15%) before randomization was higher than expected (12 of 76 enrolled participants). This was primarily due to participants moving theaters at the last minute and not being randomized in the anesthetic room. Two of these participants were withdrawn before randomization as the responsible anesthetist believed the participant was not suitable for randomization. These challenges are comparable to recent large perioperative trials^32,33^ but remains a potential source of bias as participants whose allocated operating list is changed at short notice may be different to those who proceed as originally scheduled.

We found myocardial injury and AKI were more common than previously reported regardless of treatment allocation whereas delirium was less common than expected in the control group.^5,6,34^ The incidence of AKI and myocardial injury after non-cardiac surgery (MINS) may be explained by more frequent measurements – daily renal function and troponin assessment is not routine in hip fracture care.^26^ The incidence of delirium was similar to contemporary studies.^35^ Furthermore, the regulatory approvals for the trial excluded people unable to give their own consent, therefore representing a group with lower risk of delirium. Median EQ-5D quality of life scores were comparable between groups and with previous hip fracture trials.^36^

After this pilot trial we would need to adjust the definitive trial. The composite primary outcome would remain the same. It is patient relevant, and comparable to other major hip fracture trials.^34,35,37^ However, given most events occurred within 5 days we could reduce participant and trial burden by reducing follow-up from 7 to 5 days. Targeting 2 arterial blood pressure parameters added complexity so the intervention could be rationalized to maintaining the mean arterial pressure ≥75 mm Hg throughout surgery.

Considering the generalizability of the feasibility findings, these are applicable to future trials of intraoperative hemodynamic targeting interventions in the setting of urgent surgery. Particular facets that inform future studies include web-based randomization in the anesthetic room rather than sequential envelops, and rationalization of the intervention to target 1 blood pressure variable rather than 2.

However, the statistical assumptions made in planning the pilot and definitive trial are considerably different to expectations. The absolute rates of the primary outcome were higher than expected. Moreover, the direction of effect seen in this trial was contrary to our hypothesis. Although the confidence intervals (−45% to 3%) for the difference in effect include potential benefit, the magnitude of this potential benefit is unlikely to be of clinical relevance and the size of such a study is probably unfeasible. A conservative updated power calculation, of a 5% absolute reduction in complications in the intervention group and 80% power, gives an estimated total sample size of >3000 patients. Our pilot trial suggests that anesthetists in the control arm still target hypotension avoidance as part of their usual practice. Therefore the magnitude of treatment effect from individualized blood pressure targeting versus usual care is likely to only be marginal.

In conclusion, a definitive randomized controlled trial of tight blood pressure control is technically feasible but our data are insufficient to support progressing to a definitive study.

## ACKNOWLEDGMENTS

The authors thank the research nurses of Queens Medical Centre Nottingham, Royal Sussex County Hospital Brighton, and Royal Surrey County Hospital Guildford, UK for their dedication to recruiting and following up participants.

**This manuscript was handled by:** Richard P. Dutton, MD.

## Supplementary Material
