# Supplementary material for: Hip Fracture Intervention Study for Prevention of Hypotension Trial: a Pilot Randomized Controlled Trial
Source: A A Pract. 2025 Jan 6;19(1):e01891. doi: 10.1213/XAA.0000000000001891 (PMC11761058; doi:10.1213/XAA.0000000000001891)
Supplement: Supplementary file 1 [file acc-19-e01891-s001.pdf]

Online Supporting Information for HIPHOP

Figure S1. Blood pressure during surgery by treatment allocation

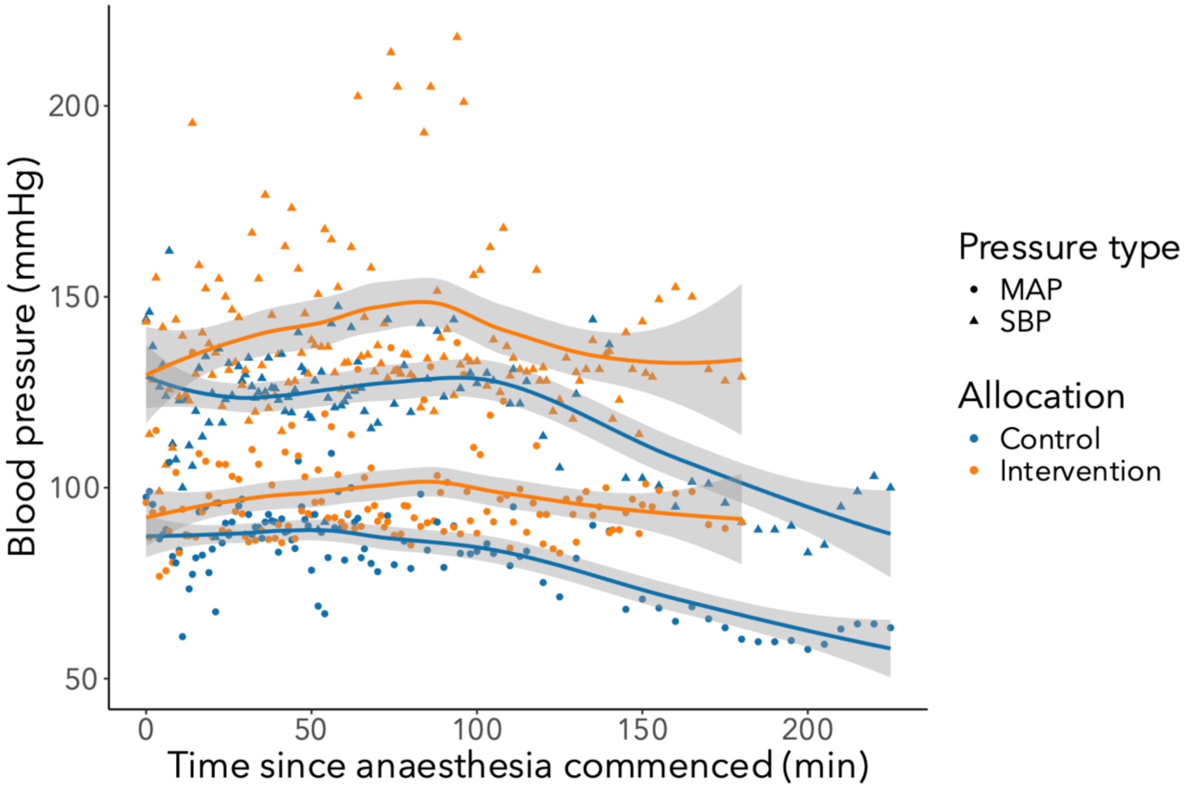

Mean blood pressure (solid lines), individual participant blood pressure (circles/triangles)

Figure S2. Lowest intraoperative blood pressures per participant

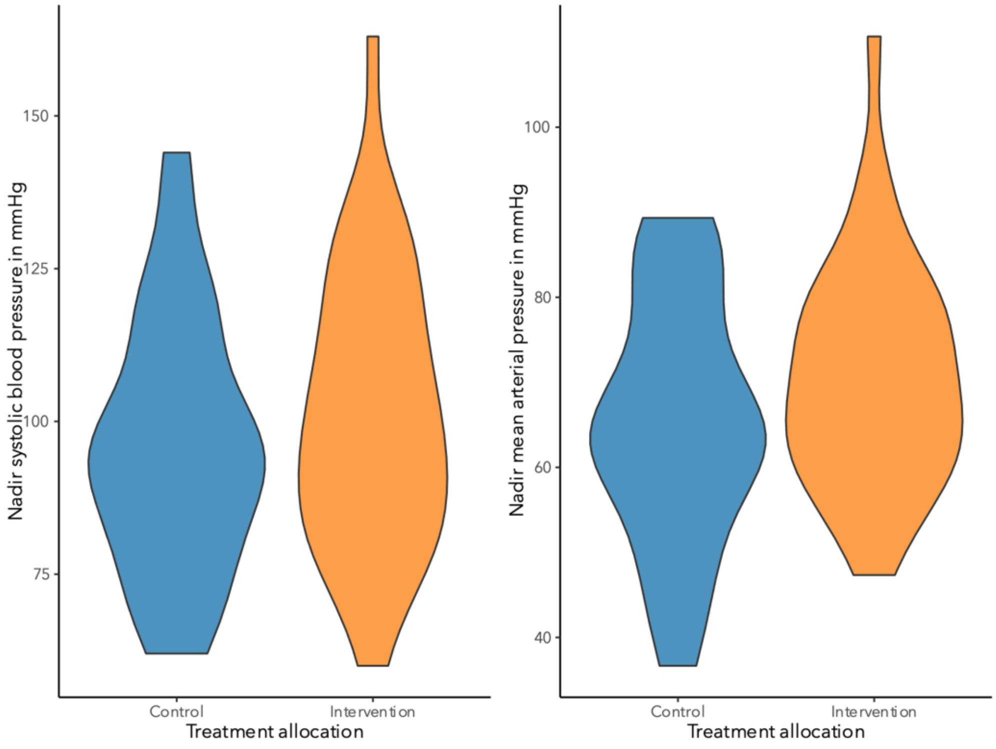

Figure S3. Total time blood pressure is below treatment threshold

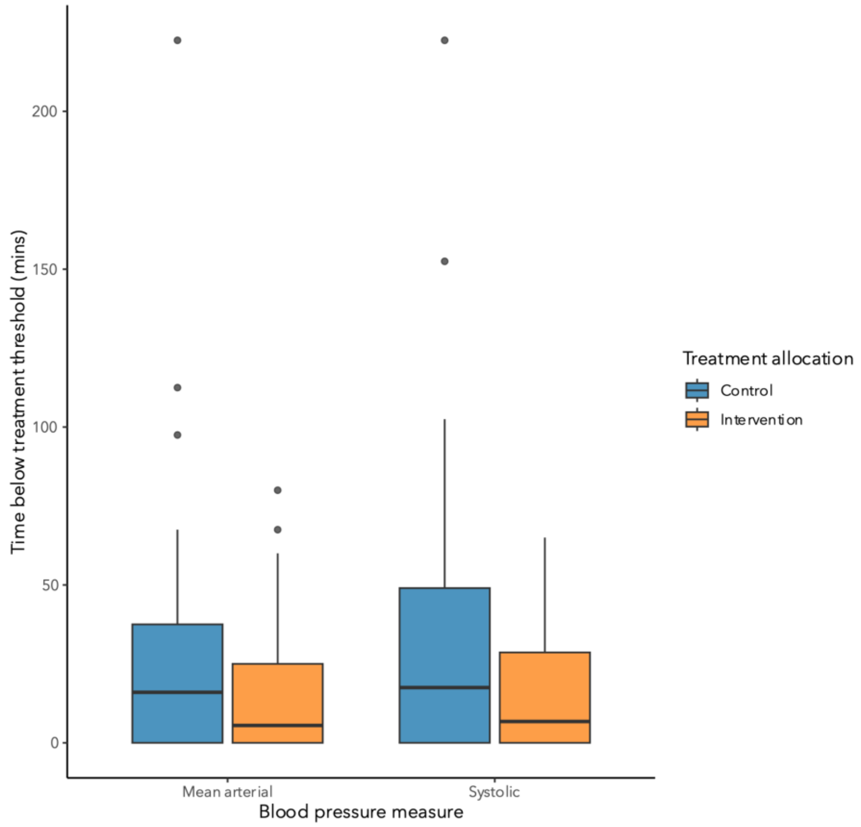

Thresholds: Systolic blood pressure <80% pre-induction, Mean arterial pressure <75mmHg.

Figure S4. Vasoactive use by treatment allocation

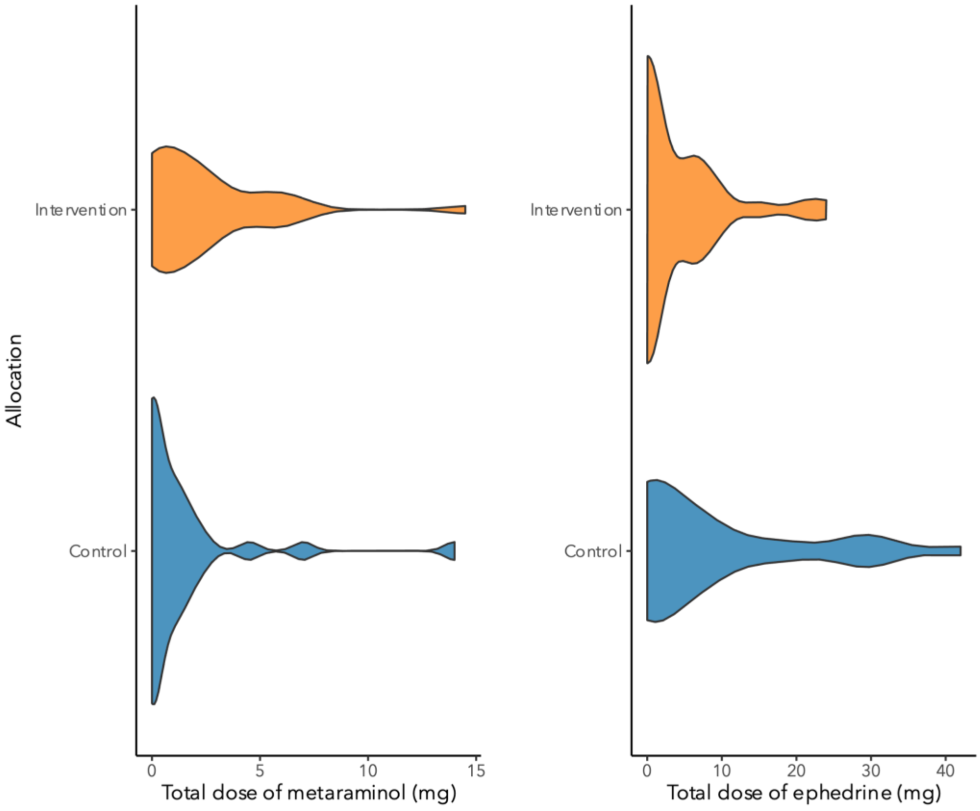

Figure S5. EQ-5D Visual Analogue Score at 30 days postoperatively for participants allocated to tight blood pressure control (orange) or usual care (blue).

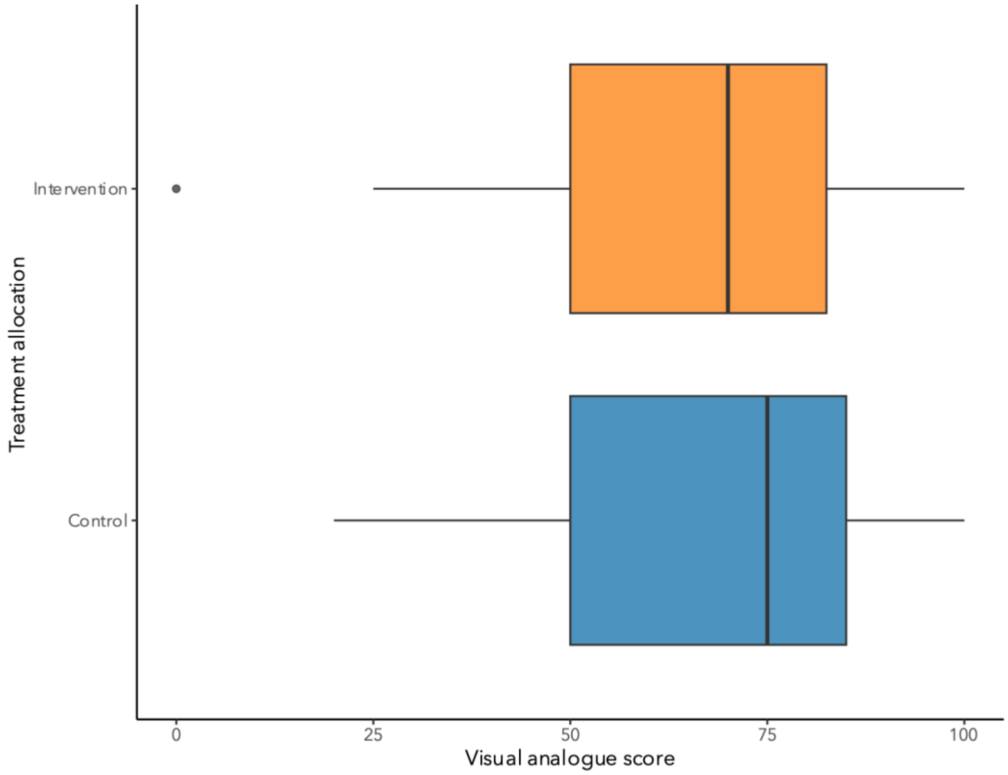

Table S1. Duration of time below treatment threshold

Threshold	Control	Intervention
Less than 80% pre-induction SBP	20 (0, 85 [0-222.5])	6 (0, 20.5 [0-65])
Less than MAP 75mmHg	18 (0, 57.5 [0-222.5])	4 (0, 25 [0-80])
Less than 80% pre-induction SBP and MAP 75mmHg	35 (0, 85 [0-222.5])	16 (2.5, 44.5 [0-80])

Median (IQR [Range]) in minutes

Table S2. Vasoactive drug administration (separating those with none given)

Total dose	**Overall**, N = 64[a]	**Control**, N = 30[a]	**Intervention**, N = 34[a]
**Metaraminol (mg)**	1.80 (1.00, 4.00 [0.10-14.50])	1.50 (1.00, 2.25 [0.10-14.00])	2.50 (1.50, 5.00 [0.30-14.50])
None used	25	15	10
**Ephedrine (mg)**	9 (6, 18 [3-42])	11 (6, 28 [3-42])	6 (6, 9 [3-24])
None used	35	14	21

[a]Median (IQR [Minimum-Maximum])

Table S3. Intraoperative fluid administration

	**Overall**, N = 64[a]	**Control**, N = 30[a]	**Intervention**, N = 34[a]
**Crystalloid (ml)**	1,000 (800, 1,000 [0-2,500])	1,000 (1,000, 1,000 [300-2,000])	1,000 (713, 1,000 [0-2,500])
**Received blood transfusion**	4 (6.3%)	2 (6.7%)	2 (5.9%)

[a]Median (IQR [Minimum-Maximum]); n (%)

Table S4. Daily postoperative creatinine and troponin values by treatment allocation

	**Troponin**		**Creatinine**	
Postoperative Day	**Control**[a]	**Intervention**[a]	**Control**[a]	**Intervention**[a]
1	95.5 ± 357.4	70.0 ± 141.2	84.8 ± 51.4	90.8 ± 44.3
2	59.7 ± 129.9	162.8 ± 438.5	85.7 ± 56.0	95.5 ± 58.4
3	37.7 ± 40.2	1,468.6 ± 7,573.0	82.3 ± 55.9	95.8 ± 59.9
4	31.5 ± 32.8	609.6 ± 2,873.5	82.3 ± 53.6	88.5 ± 56.1
5	27.3 ± 26.9	374.4 ± 1,661.9	80.0 ± 48.2	86.8 ± 50.1
6	25.6 ± 26.1	300.2 ± 1,269.9	80.8 ± 50.4	88.4 ± 45.9
7	23.5 ± 27.5	208.0 ± 771.5	78.9 ± 44.6	88.9 ± 47.3

[a]Mean ± SD

Table S5. Daily occurrence of stroke and delirium by treatment allocation

	**Stroke**		**Delirium**	
Postoperative Day	**Control**[a]	**Intervention**[a]	**Control**[a]	**Intervention**[a]
1	1 (3.4%)	0 (0%)	0 (0%)	3 (8.8%)
2	0 (0%)	0 (0%)	1 (3.3%)	2 (5.9%)
3	0 (0%)	0 (0%)	1 (3.3%)	1 (2.9%)
4	0 (0%)	0 (0%)	0 (0%)	2 (5.9%)
5	0 (0%)	0 (0%)	0 (0%)	1 (2.9%)
6	0 (0%)	0 (0%)	0 (0%)	2 (5.9%)
7	0 (0%)	0 (0%)	1 (3.3%)	2 (5.9%)

[a]n (%)

Table S6. Adverse events

**Event type**	**Control**	**Intervention**
Any adverse event	18	17
Serious adverse event	3	1
Postoperative hypotension	3	6
Anaemia	4	4
Bone cement implantation syndrome	0	0
